# Supplementary material for: Insights from COVID-19: Reflecting on the Promotion of Long-Term Health Policies in China
Source: Int J Environ Res Public Health. 2023 Feb 7;20(4):2889. doi: 10.3390/ijerph20042889 (PMC9957391; doi:10.3390/ijerph20042889)
Supplement: Supplementary file 1 [file ijerph-20-02889-s001.zip › ijerph-2170181-supplementary.pdf]

Supplementary Materials

File S1. Questionnaire.

"Healthy China 2030" is an important strategic planning outline in China and an action plan to promote the construction of a healthy China in the next 10 years, aiming to promote the construction of health-related facilities and improve the health quality of the whole people. In order to understand the implementation progress of the outline and the participation of the whole people, we have created this questionnaire. Thank you for your participation and support.

Note: Please write the option number in □ before the question according to your own situation.

The following are closed questions:

1. Gender:

①Man ②Woman

2. Age:____________

3. Living area:

①City ②Cities and towns ③Rural and village

4. Occupation category:

① Party and government organs, state organs, mass organizations and social organizations, enterprises and institutions

② Teachers and professional and technical personnel

③ Business, service industry personnel

④ Production operators

⑤ Others (students, soldiers, etc.)

5. Education level:

①Primary school ②Junior school ③Secondary or high school ④College or undergraduate (5) Graduate students and above

6. Political landscape:

①CPC member ②Member of the Communist Youth League ③The public ④Other

7. Your Health Conditions:

①Very good ②Good ③Common ④Not good ⑤Suffering from chronic diseases

8. Days when you felt uncomfortable in the past year:

①0 days ②Less than 5 days ③5-15 days ④15-30 days ⑤More than 30 days

9. Average annual income level of your family:

①Less than 80,000 yuan ②80-150,000 yuan ③150,000-300,000 yuan

④300-1 million yuan ⑤More than 1 million yuan

10. Do you think the current medical service charges are reasonable:

①Very reasonable ②Reasonable ③Common ④Unreasonable ⑤Very unreasonable

11. Total individual out-of-pocket medical expenses of all members of your family as a percentage of family income in the past year:

①Less than 1% ②1%-5% ③5%-10% ④Over 10% ⑤Unclear

12. What do you think is the current difficulty level of seeing a doctor in a large general public hospital:

①Very difficult ②Difficult ③Common ④Easy ⑤It is not difficult at all

13. How do you think the current tension in the doctor-patient relationship is:

①Very nervous ②General nervousness ③Not too nervous ④Be not nervous ⑤Very harmonious

14. How much do you know about the implementation of the "Healthy China 2030" planning objectives in China:

①Very understanding ②Basic understanding ③General understanding ④ Know little about ⑤No

15. How much do you know about assisting diagnosis and treatment in China's digital medical technologies (technologies in the fields of Internet, big data and block chain):

①Very understanding ②Basic understanding ③General understanding ④ Know little about ⑤No

16. How much do you know about assisting drug research in China's digital medical technologies (technologies in the fields of the Internet, big data, and block chain):

①Very understanding ②Basic understanding ③General understanding ④ Know little about ⑤No

17. How much do you know about Health management by digital medical technologies in China (technologies in the fields of Internet, big data and block chain):

①Very understanding ②Basic understanding ③General understanding ④ Know little about ⑤No

18. What do you know about Disease Prevention and Risk Monitoring of China's digital medical technologies (technologies in the fields of the Internet, big data, and block chain):

①Very understanding ②Basic understanding ③General understanding ④ Know little about ⑤No

19. Your understanding of smart health (medical service model that provides residents with efficient, high-quality, safe and transparent health services, continuous health information and whole-process health management):

①Very understanding ②Basic understanding ③General understanding ④ Know little about ⑤No

The following five are entitled open questions:

20. What have you done to improve your health:

①Adjust your diet ②Sports ③Health care products ④Consult a doctor ⑤Do nothing

⑥Other

21. Your focus on disease and health issues is primarily on:

①Not have ②Heart disease ③Arthritis ④Diabetes ⑤Hypertension ⑤Asthma

⑥Other

22. Through what channels do you mainly collect health-related information:

①Take a doctor's advice ②TV ③internet ④Book learning ⑤Communicate with acquaintances

⑥Other

23. What do you think are the biggest worries about medical treatment at present:

①High cost ②Appointment registration difficult ③Long queues ④Unfamiliar with process

⑤Fear of receiving error or excessive medical treatment ⑥No one to accompany

⑦Other

24. Your focus on the development of the health industry has mainly focused on:

①Health care for the aged ②Health products ③Medical tourism

④Sports Industry ⑤Pharmaceutical Industry ⑥Nursing Industry ⑦Health testing

⑧Managed care ⑨Chronic illness rehabilitation

⑩Other
